# Inorganic Scaling Mechanisms During Forward Osmosis Concentration of Fresh vs. Hydrolysed Urine: Theoretical Modelling and Experimental Validation

**DOI:** 10.3390/membranes16060197

**Published:** 2026-06-05

**Authors:** Maano Tshimange, Ṋamadzavho Enos Sitabule, Judy Lee, Siddharth Gadkari

**Affiliations:** 1 School of Chemistry and Chemical Engineering, University of Surrey, Guildford GU2 7XH, Surrey, UK; m.tshimange@surrey.ac.uk (M.T.); j.y.lee@surrey.ac.uk (J.L.); 2 Sasol Research & Technology, Sasolburg 1947, South Africa; enos.sitabule@sasol.com

**Keywords:** forward osmosis, urine, inorganic scaling, scaling reversibility, thermodynamic modelling

## Abstract

Forward osmosis (FO) offers a promising route for urine concentration and nutrient recovery, yet inorganic scaling under high water recovery remains a significant challenge. This study systematically investigated scaling during FO treatment of synthetic fresh urine (SFU) and synthetic hydrolysed urine (SHU) over three consecutive cycles to 80% water recovery. SFU exhibited moderate flux decline (~14.4 → 4–5 LMH), with minimal hydraulic resistance from sparse calcium-deficient Ca–P deposits (Ca:P ≈ 1.2; ACP/OCP-like). In contrast, SHU caused severe cumulative scaling, progressively reducing flux from 19 → 14.46 → 1.3 LMH, dominated by struvite (Mg:P ≈ 1.02) and mixed Mg–carbonate phases. Visual MINTEQ thermodynamic modelling correctly identified the dominant mineral families in both feeds, while kinetic effects governed the formation of metastable phases, demonstrating that equilibrium modelling and experimental characterisation are complementary tools for scaling prediction under transient FO conditions. Physical cleaning restored ~98–99% of water flux for both feeds, confirming that even severe SHU-induced scaling is largely hydraulically reversible. High rejection of multivalent ions (PO_4_^3−^, Mg^2+^, and Ca^2+^) was maintained throughout, confirming membrane integrity was preserved despite severe scaling. These findings demonstrate that urine hydrolysis fundamentally governs scaling pathways, severity, and reversibility in FO systems, and that simple hydraulic flushing is an effective fouling-control strategy, providing practical guidance for operating condition selection and cleaning strategy design in FO-based urine treatment applications.

## 1. Introduction

With increasing global emphasis on sustainable resource recovery and circular sanitation, human urine has gained attention as a valuable source of water and nutrients within the circular economy [1,2,3,4,5,6]. Although it accounts for less than 1% of domestic wastewater volume, urine contains about 80% of the nitrogen (N) and 50–70% of the phosphorus (P) in municipal wastewater, representing significant potential for fertiliser production [7,8]. Recovering these nutrients could offset 16–21% of agricultural N demand and 9–12% of P demand [9]. Therefore, source separation and on-site urine treatment provide a more sustainable and resource-efficient alternative to conventional mixed wastewater systems, reducing nutrient discharge to waterways while lowering the energy and water requirements of centralised treatment processes [8,10,11].

Forward osmosis (FO), an osmotically driven membrane process, has gained significant attention over the past decade for source-separated urine treatment to enable nutrient and water recovery [3,12,13]. Compared to pressure-driven processes like reverse osmosis (RO) and nanofiltration (NF), FO can offer lower energy use, greater tolerance for complex feed waters, and reduced fouling with improved reversibility [3,12,13,14]. In FO, water passes through a semi-permeable membrane driven by the osmotic pressure difference between the feed solution (FS) and a concentrated draw solution (DS). Despite these advantages, membrane fouling, particularly inorganic scaling, remains a major challenge that limits FO performance under high water recovery conditions [14,15,16,17,18].

Human urine exists in two primary forms: fresh and hydrolysed, each with distinct physicochemical characteristics [19], that strongly influence the nature and severity of inorganic scaling. Fresh urine (pH~6–7) is dominated by urea and low carbonate alkalinity, limiting immediate precipitation of calcium- and phosphate-based minerals [10,20]. Hydrolysed urine, however, undergoes urea breakdown into ammonium and carbonate ions, raising the pH (>9) and increasing ionic strength. Additional organic compounds, such as uric acid, creatine, and creatinine, further contribute to the dissolved solid load [21]. These chemical changes enhance supersaturation and promote precipitation of phosphate minerals like struvite and hydroxyapatite, particularly in the presence of calcium, magnesium, and phosphate ions [1,22]. The distinct chemical characteristics of fresh and hydrolysed urine create fundamentally different scaling environments; however, their comparative effects on FO membrane scaling under realistic operating conditions have not yet been systematically investigated.

Scaling occurs when dissolved salts concentrate at the membrane surface and exceed their solubility limits, precipitating as mineral deposits that increase hydraulic resistance, reduce water flux, increase the risk of pore blockage, and potentially accelerate membrane degradation [16,23]. Two primary nucleation pathways govern scale formation: homogeneous nucleation, in which crystals form within the bulk solution and subsequently deposit on the membrane surface; and heterogeneous nucleation, in which crystallisation initiates directly on the membrane surface, which can occur even when the saturation index (SI) is below zero due to the lower energy barrier at the solid–liquid interface [24]. Understanding which pathway dominates under a given set of operating conditions is important for predicting scaling onset and selecting appropriate mitigation strategies, and is closely linked to feed solution composition [25].

Although scaling in urine-fed FO systems has been recognised, the mechanistic understanding of scale formation, its impact on membrane performance, and the effectiveness of cleaning under transient and multicycle conditions remains limited. Existing studies have primarily focused on nutrient and water recovery performance [3,4,6], and while some have identified dominant mineral phases in urine FO systems [1], these investigations were largely confined to single fouling cycles and did not systematically assess fouling reversibility or scale progression across consecutive cycles. More broadly, existing FO scaling studies have largely focused on performance degradation indicators such as flux decline, without resolving the underlying physicochemical progression of scaling from initial surface nucleation through deposit growth to performance deterioration or systematically linking deposit characteristics to cleaning outcomes [1,14,15,16,17,18]. For example, Zhang et al. [1], the most comparable FO urine scaling study, identified dicalcium phosphate as the main precipitate in fresh urine and a mixture of struvite and hydroxyapatite in hydrolysed urine; however, they did not investigate multi-cycle scaling behaviour, cleaning reversibility, or validate thermodynamic modelling under progressively concentrated conditions.

Furthermore, while thermodynamic modelling tools such as Visual MINTEQ have been applied to predict scaling potential in related membrane systems [25,26,27,28,29,30], their predictive accuracy for urine-specific mineral phases under multi-cycle FO conditions has not been validated. This is particularly important because, as water is progressively removed, the feed composition continuously evolves, and multiple mineral phases can simultaneously become supersaturated. Despite its importance in FO-based urine treatment, a clear mechanistic understanding linking scaling progression, deposit characteristics, and cleaning reversibility under realistic multi-cycle FO conditions is still lacking, limiting the development of robust FO-based urine treatment strategies.

This study, therefore, aims to investigate the physicochemical mechanisms of inorganic scaling during multi-cycle FO concentration of fresh urine and hydrolysed, combining experimental characterisation with thermodynamic modelling. To the best of the authors’ knowledge, this is the first study to: (1) examine how transient flux decline progresses as scaling develops during the treatment of fresh and hydrolysed urine across three consecutive FO cycles at an 80% water recovery; (2) compares scaling pathways, deposit composition, and surface property changes using SEM-EDS, AFM roughness, and contact angle analyses, to provide insight into how urine hydrolysis state influences scaling behaviour, mineralogy, and cleaning reversibility; (3) to evaluate SI using equilibrium-based water chemistry for fresh and hydrolysed urine, and to experimentally validate thermodynamic predictions against observed mineral phases across progressive FO concentration stages, identifying where equilibrium modelling accurately predicts scaling and where kinetic effects dominate in this multi-supersaturated system; and (4) evaluate the effectiveness of physical cleaning in restoring membrane performance following severe cumulative inorganic scaling accumulated over three consecutive FO cycles to 80% water recovery.

## 2. Materials and Methods

### 2.1. Feed and Draw Solution Compositions

Two feed types were evaluated: synthetic fresh urine (SFU) and synthetic hydrolysed urine (SHU), prepared according to the recipes of Zhang et al. [1] and Ray et al. [30] with slight modifications. All chemicals were purchased from Sigma-Aldrich (St. Louis, MO, USA) or Fisher Scientific (Loughborough, UK). Synthetic urine was used in preference to real urine because it provides precisely controlled and reproducible ionic compositions, eliminating the batch-to-batch variability in pH, ion concentrations, and organic load inherent to real urine that would confound mechanistic attribution of scaling to urine hydrolysis state [30]. Key metabolites, including creatinine, uric acid, and hippuric acid, were included to better replicate the composition of real urine, based on human urine characterisation by Bouatra et al. [31]. The recipes of synthetic feed solutions are provided in Table S1 (Supporting Information). Sodium chloride (NaCl) at 5 M was used as the DS in all FO experiments. This concentration was selected to maximise the osmotic driving force, maintaining sufficient driving force throughout operation, even as feed osmotic pressure increased progressively with water removal toward 80% recovery [32]. The volumes of the FS and DS were 5 and 3 L, respectively. A summary of ionic compositions is presented in Table 1.

### 2.2. FO Membrane

For all experiments in this work, a commercial flat-sheet FO membrane made from cellulose triacetate (CTA), supplied by Fluid Technology Solutions (FTS H2O, Albany, OR, USA), was employed.

### 2.3. Experimental Setup

**Figure 1:**
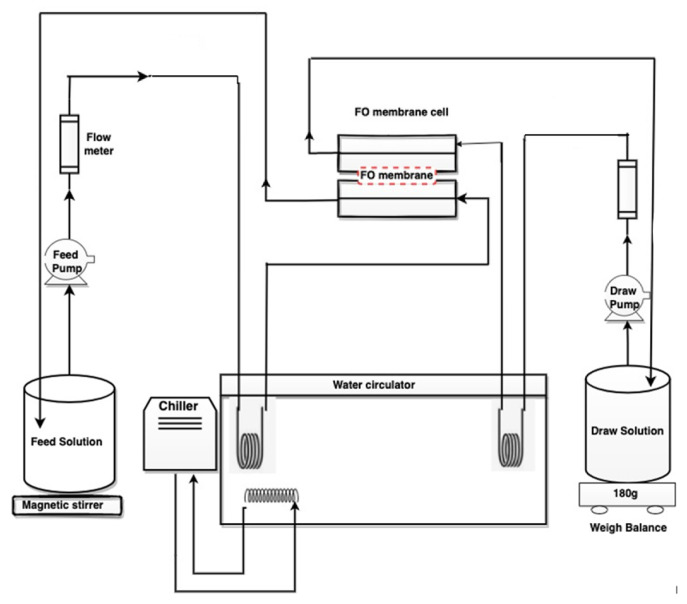
Schematic diagram of the FO system used in the study. The system comprises a feed tank mounted on a magnetic stirrer, a temperature-controlled water bath connected to a chiller, and gear pumps that circulate feed and draw solutions through the FO membrane cell in counter-current mode. DS mass was continuously recorded using a digital balance to determine water flux.

The experiments were carried out on a custom-built FO bench-scale rig (Figure 1). The system consisted of an FO membrane cell with two identical flow channels (145 mm × 95 mm × 1.5 mm), for an effective membrane area of 140 cm^2^. Two variable-gear pumps (Micropump Inc., Vancouver, WA, USA) were used to continuously circulate the FS and DS through each flow channel. All experiments were conducted in the active-layer-facing-feed-solution (AL-FS) orientation, whereby the active layer faced the FS and the support layer faced the DS. Temperature was maintained at 20 °C using a temperature controller with submergible stainless-steel coils. A magnetic stirrer was used to maintain the homogeneity, while the DS tank was placed on a digital mass balance (Kern CB, Balingen, Germany) connected to a computer to record the DS weight change over time and determine the water flux. The crossflow cell will be operated in counter-current mode, with a CFV of 0.15 m/s for both DS and FS. Conductivity and pH of the FS were measured regularly using a calibrated conductivity/pH metre (Mettler-Toledo, Columbus, OH, USA).

The water flux was determined by calculating the change in the FS volume over time, normalised by the membrane surface area. It was calculated using Equation (1):

(1) $$J_{w}=\frac{V_{ft1}-V_{ft2}}{A_{m}∆t}$$

where *J_w_* is the water flux (LMH), *V*_*ft*1_ and *V*_*ft*2_ are the initial and final FS volumes (L), *A_m_* is the effective membrane area (m^2^), and Δt is the duration of the experiment (h).

### 2.4. Experimental Procedure

Before each experiment, FO membranes were pre-soaked in deionised water (DI) for at least 24 h to remove preservative residues, followed by a 30 min equilibration step using DI water on both FS and DS sides to establish steady-state flux. A baseline test was then conducted using DI water as the FS and 5 M NaCl as the DS to characterise clean membrane performance. Subsequently, the feed tank was filled with 5 L of urine solution and the DS tank with 3 L of 5 M NaCl. Three consecutive fouling cycles were performed using the same FO membrane without intermediate cleaning to capture the progressive accumulation of scaling deposits across cycles. Each cycle was terminated when water recovery reached 80%, after which the concentrated FS was discarded and replaced with 5 L of fresh urine solution, and the diluted DS was discarded and replaced with 3 L of fresh 5 M NaCl, fully restoring the osmotic driving force for the subsequent cycle. Following completion of all three fouling cycles, a post-fouling flux test was first conducted by circulating DI water on both sides of the membrane for 1 h at the operating CFV (0.15 m/s), to quantify the flux decline attributable to the accumulated fouling layer under low-driving-force conditions. Physical cleaning was then performed by flushing both sides of the membrane with DI water at twice the operating CFV (0.3 m/s) for 20 min, to assess fouling reversibility and calculate flux recovery.

### 2.5. Characterisation of Precipitate

#### 2.5.1. Scanning Electron Microscopy (SEM) with Energy Dispersive X-Ray Spectroscopy (EDS)

The morphology of the precipitate was observed with SEM-EDS (Thermo Scientific Apreo 2). Membrane coupons were air-dried overnight and mounted onto aluminium stubs with double-sided copper tape. Before imaging, a thin gold coating was applied to improve electrical conductivity. SEM analysis was performed at 15 kV and at various magnifications to capture detailed images of fouling deposits and membrane surface features. Concurrently, elemental analysis of the foulants was conducted using an EDS detector, providing insights into the chemical composition of the deposits.

#### 2.5.2. Membrane Roughness Analysis

The surface morphology and roughness of the membranes were characterised using atomic force microscopy (AFM) (Bruker, Billerica, MA, USA) operated under ambient conditions. A silicon probe was used for imaging after air-drying the membrane samples to minimise moisture interference. To obtain representative roughness parameters, at least 3 measurements were performed on each membrane specimen, ensuring data reproducibility.

#### 2.5.3. Contact Angle Analysis

The hydrophobicity and hydrophilicity of the fouled membranes were quantitatively assessed by measuring the water contact angle (CA) using the sessile drop technique with a KRÜSS DSA30 goniometer (Hamburg, Germany). Membrane samples, dried for 24 h, were mounted on pre-cleaned glass slides using double-sided adhesive tape. A 2 μL droplet of deionised water was deposited onto the membrane surface, and CAs were recorded at a minimum of five distinct locations on each specimen. The mean CA was calculated and reported as a measure of surface wettability.

### 2.6. Analytical Methods

All the samples were filtered through a 0.45 μm syringe filter before analysis. The quantification of major inorganic ions (SO_4_^2−^, PO_4_^3−^, Na^+^, K^+^, Ca^2+^, Mg^2+^, and Cl^−^) was carried out using ion chromatography (IC, Dionex ICS-5000; Thermo Scientific, Waltham, Massachusetts, USA). Samples collected from both synthetic urine and draw solutions were appropriately diluted before analytical measurement. For instrument calibration, external standard solutions containing 1000 ppm of each element, obtained from Fisher Chemicals Supplier, UK, were prepared by serial dilution with deionised water to achieve a range of concentrations (0.1, 1, 5, 10, 20, and 50 mg/L). Calibration curves were constructed to establish correlations between the peak heights or areas detected for each analyte and their corresponding concentrations in both urine and DS samples.

The membrane rejection rate (R) was determined according to the equation below [33]:

(2) $$R\;\left(\%\right)=1-\frac{\mathrm{DF}\times \;C_{D}}{C_{F}}\;\times \;100$$

where the dilution factor (DF) is defined as:

$$DF=\frac{V_{D}}{V_{P}}$$

In these expressions, $C_{D}$ represents the final concentration of contaminants in the DS (mg/L), and $C_{F}$ denotes the initial concentration of contaminants in the FS (mg/L). $V_{P}$ is the permeate volume (L) transferred from the FS to the DS, and $V_{D}$ is the final volume of the DS (L).

### 2.7. Theoretical Prediction of Inorganic Scaling Formation

Visual MINTEQ (version 4.0, KTH, Sweden) was used to calculate ion speciation, concentrations, and solubility in aqueous solutions [29,30]. Predicting the potential for mineral precipitation (scaling) during water removal from SFU and SHU. The analysis examined variations in the SI of selected mineral phases with increasing concentration factors (CF). Negative SI values (SI < 0) indicate undersaturation, SI = 0 denotes equilibrium, and positive SI values (SI > 0) reflect supersaturation conditions that favour spontaneous precipitation [34]:

(3) $$SI={log}_{10}(\mathrm{IAP})+pK_{sp}$$

where IAP represents the ionic activity product, and *pK*_sp_ is the negative logarithm of the solubility product (*K*_sp_).

The ion dissolution/association model in Visual MINTEQ predicts scaling potential based on solution chemistry. The Specific Ion Interaction Theory (SIT) was employed to account for high ionic strengths (up to 4 M) [35,36], assuming thermodynamic equilibrium, binary ion–ion interactions for activity corrections, constant temperature, and homogeneous mixing. Kinetic effects, concentration polarisation, and surface-specific nucleation were not considered. Simulations used the ionic compositions from.

Table 1, with pH 6.0 for SFU and pH 9.0 for SHU at 20 °C, assuming full ion dissociation, default complexation reactions, and chemical equilibrium.

## 3. Results and Discussion

### 3.1. Water Flux Performance During Multiple Filtration Cycles

**Figure 2:**
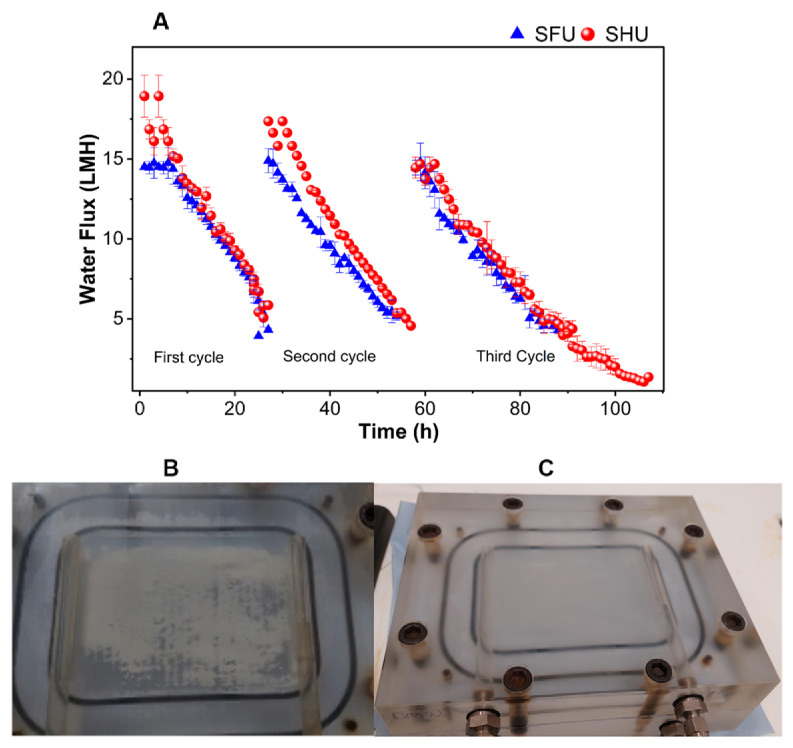
(**A**) Water flux profiles of SFU and SHU during three filtration cycles; (**B**) Membrane surface after the final SHU cycle, showing extensive fouling coverage; (**C**) Membrane surface after the final SFU cycle, displaying negligible fouling.

Figure 2A presents the water flux evolution of SFU and SHU over three consecutive cycles conducted without intermediate cleaning to assess progressive fouling accumulation. SHU showed a higher initial flux (19 LMH) than SFU (14.43 LMH) despite its higher bulk feed osmotic pressure (~23 bar vs. ~13 bar). With a 5 M NaCl draw solution providing an overall driving force of ~270 bar, the difference in feed osmotic pressure accounts for less than 4% of the total driving force and is therefore insufficient to explain the observed flux behaviour. This contrasts with Zhang et al. [1], who reported comparable or lower flux for hydrolysed urine; however, in that study, weaker draw solutions (0.5–2 M NaCl) were used, in which the osmotic pressure difference between fresh and hydrolysed urine represented a substantially larger fraction of the total driving force. Under the high-driving-force conditions of the present study, feed ion diffusivity becomes the dominant factor governing initial flux behaviour. Instead, the higher flux in SHU is attributed to its dominant ionic species, NH_4_^+^ and HCO_3_^−^ formed during urea hydrolysis, which have significantly higher diffusivity than the urea, Ca^2+^, and Mg^2+^ present in SFU. In the AL-FS orientation used in this study, the DS (5 M NaCl) faces the support layer and experiences dilutive internal concentration polarisation (ICP), identical across both SFU and SHU experiments, since the same DS concentration was used throughout. The flux difference between SFU and SHU is therefore governed by external concentration polarisation (ECP) on the feed side, where feed ion diffusivity determines the rate at which accumulated solutes back-diffuse away from the active layer surface, thereby preserving the effective osmotic driving force. As shown by Volpin et al. [37], ion diffusivity plays a key role in governing concentration polarisation and water flux in FO systems. NH_4_^+^ and HCO_3_^−^, the dominant species in SHU, have diffusivities roughly an order of magnitude higher than urea, which dominates SFU, resulting in lower ECP at the membrane surface and a higher effective osmotic driving force, thereby explaining the higher initial flux of SHU. However, this elevated flux also intensifies the concentration polarisation of scaling-prone species, accelerating supersaturation and contributing directly to the more severe scaling observed in SHU.

During cycles 1 and 2, SFU showed a consistent flux decline to ~4–5 LMH at 80% recovery, indicating that reductions were mainly driven by DS dilution and feed concentration rather than scaling [38,39]. In cycle 3, the initial flux decreased only slightly (~14 LMH) and declined to ~4 LMH, confirming the absence of significant inorganic scaling. The near-neutral pH of SFU (≈6.0) limits precipitation of minerals such as struvite and calcium phosphate, and the stable flux behaviour across all cycles implies minimal scaling accumulation.

Contrastingly, SHU performance progressively declined. The initial flux decreased from 19 LMH (cycle 1) to 14.46 LMH (final cycle), while the terminal flux dropped to 1.3 LMH at 80% recovery, indicating severe cumulative inorganic fouling. This decline reflects progressive deposit accumulation on the membrane, as the same membrane was used continuously across all three cycles, meaning each successive cycle began with residual deposits from the previous run, increasing hydraulic resistance and reducing the effective osmotic driving force from the outset. This behaviour aligns with SHU’s higher ionic strength (~0.4 M vs. ~0.17 M in SFU), alkaline pH (9.0 vs. 6.0), and the presence of hydrolysis products such as ammonium bicarbonate (Table S1). Elevated pH shifts phosphate speciation toward HPO_4_^2−^ and PO_4_^3−^ and increases CO_3_^2−^ concentrations, promoting CaCO_3_ and Ca/Mg-phosphate supersaturation at the membrane interface despite lower bulk divalent cation levels [40]. These mechanistic effects explain the near-complete flux suppression (1.3 LMH) by the third cycle in SHU, whereas SFU remained stable throughout. Overall, the results confirm that the urine hydrolysis state strongly governs inorganic scaling severity in FO systems, with hydrolysed urine promoting far greater flux decline and deposit accumulation than fresh urine. This is supported by membrane images (Figure 2B,C), which show dense fouling on SHU membranes and minimal deposition on SFU membranes.

### 3.2. Ion Rejection Performance

**Figure 3:**
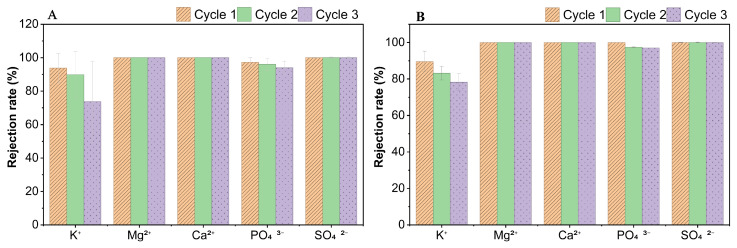
Rejection rates of ions during three consecutive concentration cycles of FO treatment of (**A**) SFU and (**B**) SHU.

The rejection performance of the major ions during three consecutive FO concentration cycles of SFU and SHU is shown in Figure 3. Overall, the membrane maintained high selectivity throughout multi-cycle operation. Divalent cations (Mg^2+^ and Ca^2+^), PO_4_^3−^, and SO_4_^2−^ were consistently well-retained across all cycles, with Mg^2+^ and Ca^2+^ rejection approaching ~100%, PO_4_^3−^ rejection remaining high at 94–97%, and SO_4_^2−^ rejection maintained at ~99% in both feed types.

These findings are consistent with previous FO studies on urine concentration reporting >95% rejection of PO_4_^3−^ and near-complete exclusion of Mg^2+^ [1,41]. The strong retention of multivalent ions can be attributed to electrostatic repulsion between the negatively charged CTA membrane surface and anionic species such as PO_4_^3−^ [42], as well as steric hindrance associated with their larger hydrated radii [41]. The sustained near-complete rejection of Mg^2+^ and Ca^2+^ across all three cycles suggests that the CTA membrane active layer retained its structural integrity despite progressive scaling accumulation, consistent with the reversible nature of inorganic fouling under osmotically driven conditions.

Potassium, however, exhibited comparatively lower rejection and a clear decline over successive cycles. Initial K^+^ rejection (90–93%) decreased to 74% in SFU and 78% in SHU by Cycle 3. This behaviour is consistent with its smaller hydrated radius relative to the effective membrane pore size (3–5 Å) [43], which facilitates greater diffusive transport toward the DS. Similar K^+^ rejection ranges (39–71%) have been reported in FO urine studies [41]. The progressive decline in K^+^ rejection with increasing cycle number is likely associated with elevated ionic strength and enhanced charge shielding at higher concentration factors, which reduce electrostatic exclusion and promote monovalent ion transport.

### 3.3. Composition and Morphology of the Scales

#### 3.3.1. SEM-EDS Analysis

**Figure 4:**
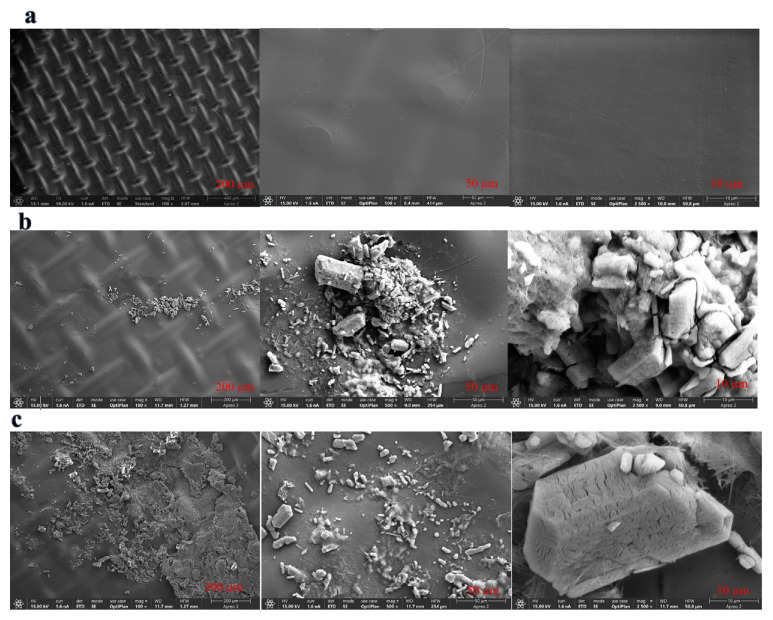
SEM images of: (**a**) Pristine membrane, (**b**) scaled FO membrane with active surface facing SFU, and (**c**) scaled FO membrane with active surface facing SHU, at different magnifications (100×, 500× and 2500×).

SEM was used to examine the surface morphology of the fouled membranes and gain insight into the scaling behaviour. Figure 4a shows the pristine membrane, while Figure 4b,c, shows membranes fouled by SFU and SHU, respectively. The SFU membrane showed only sparse coverage, while the SHU membrane was almost completely coated, indicating much more extensive scaling. This observation aligns with the transient flux decline shown in Figure 2A, confirming that the flux decline in SHU resulted from severe scaling. As concentration increased, scaling spread progressively across the membrane surface, with bulk crystallisation in the feed solution further exacerbating surface deposition, eventually suppressing flux to near-zero [44,45].

#### 3.3.2. Elemental Composition

**Figure 5:**
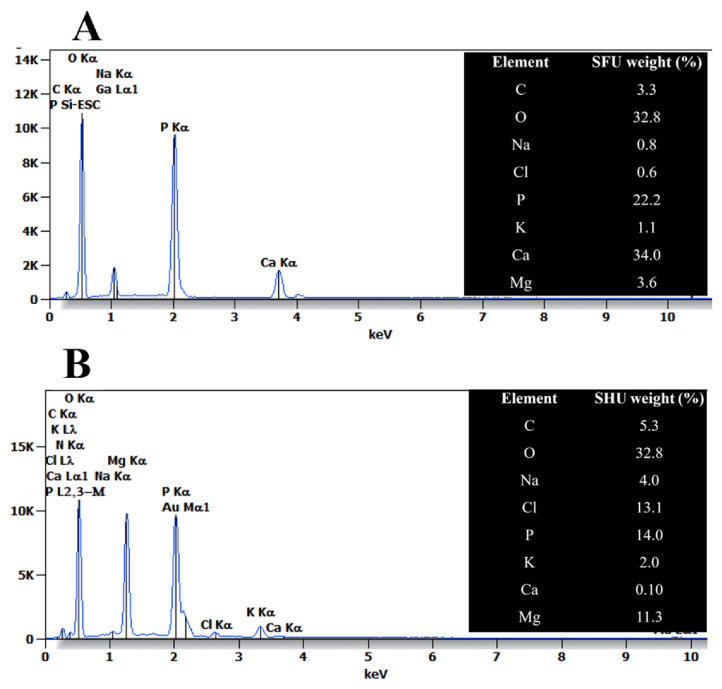
EDS analysis of the elemental composition of the scalants detected on the membrane surface after (**A**) SFU and (**B**) SHU treatment.

The elemental composition of scale deposits on the membrane surfaces was determined by EDS. For the SFU-fouled membrane (Figure 5A), major elements included C (3.3%), O (32.8%), P (22.2%), and Ca (34%), with minor contributions from Na (0.8%), Cl (0.6%), K (1.1%), and Mg (3.6%). The low Na and Cl signals are attributed to residual urine salts or trace NaCl draw solution carryover rather than discrete mineral phases. The Ca:P atomic ratio of ~1.20 indicates a calcium-deficient calcium phosphate, consistent with amorphous calcium phosphate (ACP), poorly crystalline octacalcium phosphate (OCP), or a mixture of brushite and other Ca-rich phases [46]. Meanwhile, the SHU-fouled membrane (Figure 5B) was dominated by C (5.3%), O (32.8%), Na (4%), Cl (13.1%), Mg (11.3%), and P (14%), with only trace Ca (0.10%). The high Mg and P content, with an Mg:P ratio of ~1.02, strongly supports struvite precipitation, in agreement with previous studies on hydrolysed urine [47], thereby confirming its precipitation on the membrane surface. Similar Mg:P ratios and orthorhombic morphologies have been reported in FO membranes treating hydrolysed urine [12,48]. Additionally, the detection of a K peak (2%) suggests the possible co-precipitation of potassium struvite (K-struvite, MgKPO_4_·6H_2_O) [49], alongside ammonium struvite.

The EDS findings are consistent with the flux decline observed behaviour in Figure 2. For SFU, the sparse Ca–P deposits identified are consistent with limited scaling accumulation, explaining the relatively stable flux profiles across cycles. For SHU, the dense struvite-dominated layer accounts for the progressive and severe flux suppression observed by the third cycle. Together, the EDS data confirm that the urine hydrolysis state governs not only the extent but also the mineralogical character of scaling in FO systems.

### 3.4. Mineral Precipitation Prediction

**Figure 6:**
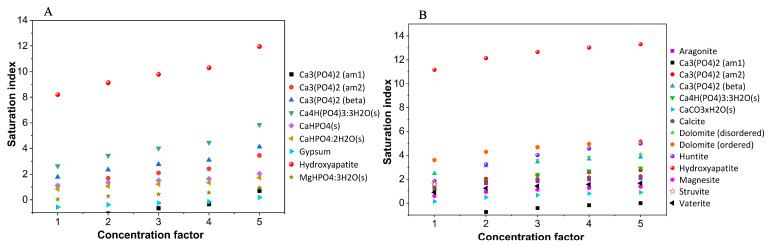
SI calculated with Visual MINTEQ software (version 4.0, KTH, Stockholm, Sweden) for potential mineral scales in (**A**) SFU and (**B**) SHU across different concentration factors. SI > 0 → precipitants formed.

In the previous section, through experimental analysis, the scaling formation, extent of it and the scalants on the membrane surface and FO performance reduction were analysed. However, for optimal process operation, it is important to predict scaling even when the ionic composition of urine varies. Therefore, to complement the FO experiments, Visual MINTEQ was employed to assess the thermodynamic propensity for mineral precipitation during progressive concentration of SFU and SHU, with predictions validated against experimentally observed mineral phases. SI and supersaturation were theoretically calculated to predict the feed concentration at which nucleation initiates. These predictions were validated by comparing them with experimentally measured flux decline due to scaling. Figure 6 compares the SI and S profiles for key candidate minerals during SFU (Figure 6A) and SHU (Figure 6B) concentration, while the overall predicted SI values are summarised in Tables S2 and S3.

Visual MINTEQ modelling indicates that calcium phosphate phases are the main supersaturated species during SFU concentration (Figure 6A). Hydroxyapatite (HAP, Ca_5_(PO_4_)_3_OH) exhibits the highest SI values across all concentration factors (CF), increasing steadily from 8.20 at CF1 to 10.30 at CF5, confirming its strong thermodynamic stability [45,46]. Its very low solubility suggests that HAP is likely to precipitate first, limiting the availability of calcium and phosphate for other phases [45]. Other calcium phosphate phases, including β- and amorphous Ca_3_(PO_4_)_2_, dicalcium phosphate dihydrate (DCPD, Ca_4_H(PO_4_)_3_·3H_2_O), and dicalcium phosphate polymorphs, also increase in supersaturation, reflecting the progressive accumulation of calcium and phosphate and their contribution to scaling. Because the SFU pH is 6, magnesium-containing minerals (MgHPO_4_·3H_2_O) remain only slightly supersaturated, since substantial Mg-phosphate precipitation only occurs at higher pH values (≥7) [50]. Critically, minerals irrelevant to the SFU system, including struvite (SI: −14.80 to −13.97) and calcite (SI: −14.94 to −14.27), remain deeply undersaturated across all concentration factors, confirming that scaling in SFU is exclusively driven by Ca–P mineralogy. The results confirm that calcium phosphate, particularly HAP, is the principal inorganic fouling risk during SFU concentration. Experimentally, however, SEM–EDS of the fouled membranes showed Ca–P deposits with Ca:P ratios below the stoichiometric value of HAP (1.67), more consistent with calcium-deficient amorphous calcium phosphate (ACP) and/or poorly crystalline OCP [46]. Despite HAP being thermodynamically favoured according to SI calculations, it was not detected experimentally. This discrepancy between the thermodynamically predicted phase (HAP) and the experimentally observed phases (ACP/OCP) reflects the kinetic limitations of Ca-P nucleation: HAP, though the most stable phase, nucleates slowly, whereas metastable phases such as ACP and OCP form readily under moderate supersaturation and near-neutral to slightly acidic conditions [46,51,52]. Over longer timescales, these metastable phases can transform into HAP [53]; however, within the limited duration of the FO experiments, kinetic control dominates, explaining why metastable Ca-P phases are observed despite the strong thermodynamic favourability of HAP. This highlights a limit of the model’s predictive capability: equilibrium thermodynamics can identify the dominant mineral family, but not the specific polymorph formed under real FO conditions.

In SHU, the scaling prediction differed from SFU due to compositional changes. Visual MINTEQ modelling (Figure 6B) indicates that a broad range of calcium phosphate and carbonate phases dominate supersaturation during SHU concentration. HAP exhibited substantially higher SI values than in SFU, increasing from 11.17 at CF1 to 13.31 at CF5, reflecting the combined effect of alkaline pH (9.0), elevated phosphate speciation toward deprotonated PO_4_^3−^, and higher ionic strength [54]. Carbonate minerals, including calcite (SI: 1.47 → 2.24), aragonite (SI: 1.32 → 2.09), vaterite, hydrated calcium carbonate, dolomite ordered (SI: 3.60 → 5.15) and disordered, and huntite (SI: 1.86 → 4.98), also remain supersaturated due to increased carbonate availability from urea hydrolysis [54,55,56,57,58]. The thermodynamic favourability of dolomite and huntite under these conditions is consistent with a sequential nucleation pathway in which Mg-calcite first precipitates in the presence of calcite and Mg^2+^, forming a surface for the subsequent formation of dolomite and then huntite [9]. Although this sequence was not directly confirmed in the present FO experiments, which used SEM-EDS rather than XRD for phase identification, it provides a plausible explanation for the sustained supersaturation of these phases predicted by the modelling at higher concentration factors. Notably, struvite exhibited a sharp and dramatic transition, supersaturated at CF1 (SI: +1.59), but dropping abruptly to deeply undersaturated conditions by CF2 (SI: −13.31) and remaining so through CF5 (SI: −12.71). This behaviour arises because the high alkalinity of SHU promotes the formation of stable magnesium–carbonate complexes that effectively compete with and limit free Mg^2+^ availability for struvite formation [59], and this transition occurs rapidly between CF1 and CF2, rather than gradually, indicating that the window of struvite supersaturation is narrow and confined to the very early stages of concentration.

Experimentally, SEM-EDS identified struvite as the dominant crystalline phase on SHU-fouled membranes, apparently contradicting the modelling prediction that struvite becomes undersaturated at higher concentration factors. This apparent discrepancy is reconciled by the transient nature of struvite supersaturation: struvite forms readily in the early stages of concentration when Mg^2+^ and NH_4_^+^ are both freely available, and supersaturation conditions are favourable. Once the system reaches higher concentration factors and magnesium-carbonate complexation intensifies, struvite formation is thermodynamically suppressed; however, the deposits already formed at early stages persist on the membrane surface and dominate the final EDS signal. This sequence highlights the importance of kinetic and temporal factors in governing which phases [60,61] ultimately accumulate on the membrane and underscore the limitation of equilibrium-based modelling for predicting deposit composition under transient FO operating conditions.

Overall, Visual MINTEQ modelling revealed distinct, hydrolysis-dependent scaling pathways during FO concentration of SFU and SHU. In SFU, calcium phosphate phases dominated supersaturation, with HAP exhibiting the highest SI values. In contrast, SHU displayed broader scaling pathways, characterised by HAP SI values exceeding 13, sustained carbonate supersaturation, and a shift in struvite from supersaturation to undersaturation as magnesium–carbonate complexation intensified. The modelling correctly identified the dominant mineral families observed by SEM–EDS (Ca–P in SFU and Mg–P/carbonate in SHU). However, the actual deposit composition was governed by kinetic constraints: metastable Ca–P phases formed instead of HAP in SFU, while struvite dominated early SHU deposits despite magnesite becoming thermodynamically favoured at higher concentration factors. These discrepancies demonstrate that modelling and experimental characterisation are complementary, with the former predicting dominant mineral families, whereas the latter resolves the specific phases formed under kinetic and interfacial FO conditions.

### 3.5. Contact Angle and AFM Analysis

**Figure 7:**
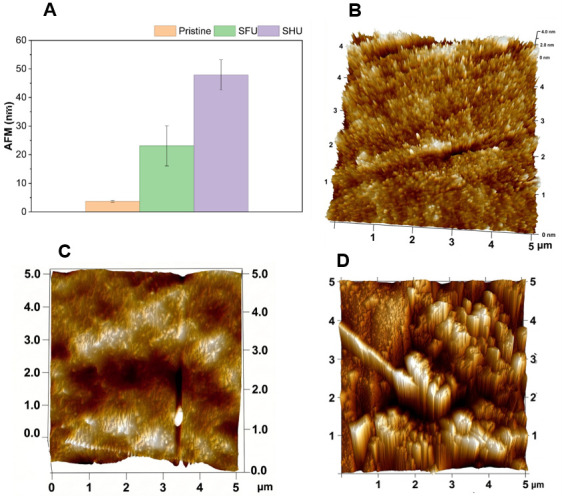
(**A**) Quantitative surface roughness analysis of pristine and fouled membranes, together with representative 3D surface topography images of (**B**) the pristine membrane, (**C**) the membrane fouled with SFU, and (**D**) the membrane fouled with SHU.

AFM and contact angle analyses were conducted to characterise how scaling deposit accumulation altered the membrane surface topography and wettability, and to assess the implications of these changes for deposit adhesion. The AFM images revealed distinct changes in surface topography between the pristine and fouled membranes, as shown in Figure 7. The pristine membrane exhibited a relatively smooth surface with a mean roughness (Ra) of 3.66 ± 0.30 nm, consistent with values reported for CTA FO membranes in previous studies [62,63,64]. In contrast, membranes exposed to SFU and SHU (Figure 7C,D) showed noticeable changes in morphology, characterised by small ridge-and-valley structures. Surface roughness increased substantially, increasing from 3.66 ± 0.30 nm in the pristine membrane to 23.10 ± 7 nm and 47.90 ± 5.28 nm for SFU- and SHU-fouled membranes, respectively. The substantially higher roughness of the SHU-fouled membrane aligns with the denser, thicker struvite-dominated deposits identified by SEM–EDS, which produce a more irregular surface topography compared to the relatively sparse Ca–P deposits formed under SFU conditions.

**Figure 8:**
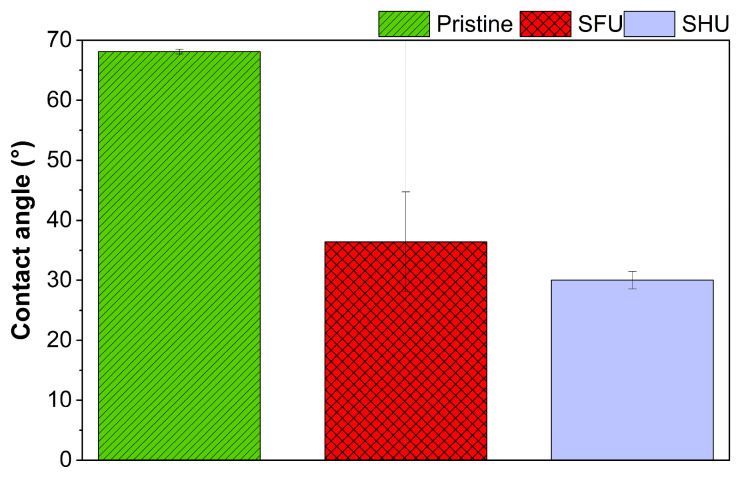
Water contact angles of the CTA FO membrane in pristine, FU-fouled, and SHU-fouled conditions. Data are presented as mean ± standard deviation.

Contact angle (CA) measurements were conducted alongside the AFM analysis to evaluate how fouling altered the membrane’s surface wettability (Figure 8). The pristine membrane exhibited a CA of 68.09°, consistent with its moderately hydrophilic character [65,66]. Following exposure to the synthetic urine feeds, the CA decreased markedly to 36.38° for the SFU-fouled membrane and 30.01° for the SHU-fouled membrane.

This substantial reduction in CA reflects the combined influence of changes in surface chemistry and increased surface roughness. According to Wenzel’s model, roughening an already hydrophilic surface enhances its wettability by promoting greater water spreading and therefore lowering the apparent CA [67]. The AFM results showed clear increases in roughness for both fouled membranes, suggesting that roughness-induced amplification of hydrophilicity contributed significantly to the observed CA decline [68,69,70]. However, the decrease in CA should not be interpreted as evidence that the foulants themselves are intrinsically more hydrophilic than the pristine membrane. Instead, the wetting behaviour likely arises from the combined effects of (i) surface roughening, (ii) the hydrated nature of the fouling layer, and (iii) the presence of inorganic precipitates, particularly phosphate- and ammonium-containing species in SHU that retain water and enhance surface water affinity. The greater CA reduction for SHU reflects its thicker fouling layer and higher content of hydrophilic inorganic phases. The low CA indicates a persistent hydration shell, allowing water to penetrate the mineral cake, unlike hydrophobic organic foulants that adhere strongly to CTA supports.

### 3.6. Flux Reversibility by Physical Cleaning

**Figure 9:**
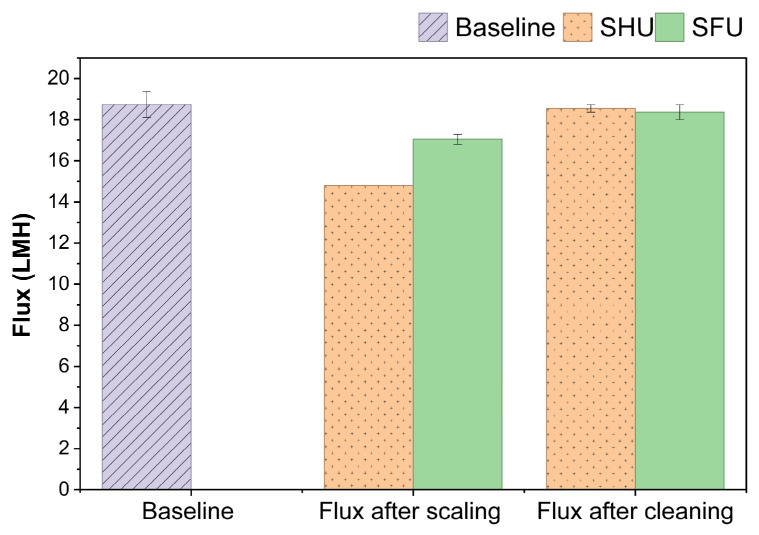
Water flux of FO membranes before fouling (baseline), after three fouling cycles without cleaning, and after physical cleaning by surface flushing.

Scaling not only degrades membrane performance but can also damage the membrane itself. To evaluate cleaning effectiveness, the flux recovery rate was measured after the final cycle following membrane cleaning using high-shear conditions generated by increased crossflow velocity. Figure 9 compares the average water flux of the pristine membrane, the flux after scaling deposition, and the flux recovery after cleaning. The initial flux of the pristine membrane was 18.74 LMH. After three cycles without intermediate cleaning, the flux decreased to 17.04 LMH (~9% decline) for SFU and 14.8 LMH (~21% decline) for SHU, reflecting the greater cumulative scaling accumulation under hydrolysed urine conditions, consistent with the denser and more extensive scalant accumulation shown in the previous SEM-EDS and visual analysis. Although SHU resulted in more severe flux decline during operation (down to 1.3 LMH by the third cycle), the post-fouling flux recovery (14.8 LMH) indicates that the deposits did not cause irreversible pore blockage under the studied conditions. Physical cleaning by high crossflow flushing (0.3 m/s) effectively restored membrane permeability, achieving flux recoveries of ~98% for SFU and ~99% for SHU.

These results confirm that inorganic scaling under these conditions is largely hydraulically reversible, regardless of the type of deposits accumulated on the membrane surface, and this behaviour is attributed to the absence of compressive hydraulic pressure in FO, which limits irreversible compaction of deposits into the membrane structure [24]. In addition, the hydrophilic nature of the inorganic deposits, as indicated by low contact angles (Figure 8), facilitates water penetration at the deposit–membrane interface during flushing, thereby promoting detachment. From a process perspective, fouling in FO is therefore dominated by surface accumulation rather than irreversible internal blockage under the conditions studied. This implies that effective end-of-cycle cleaning is sufficient to restore performance. However, predicting scaling onset, progression, and dominant scalant species through supersaturation analysis and modelling remains essential for optimising operation and managing cumulative deposit formation across FO cycles. In real urine treatment systems, the presence of organic matter and mixed foulants is expected to increase adhesion strength and cleaning complexity compared to the inorganic scaling investigated here.

## 4. Conclusions

This study systematically investigated inorganic scaling mechanisms during multi-cycle FO concentration of SFU and SHU to 80% water recovery, demonstrating that the urine hydrolysis state fundamentally governs inorganic scaling pathways, severity, and reversibility. Fresh urine limits mineral precipitation, whereas hydrolysed urine, with its alkaline pH, elevated ionic strength, and high NH_4_^+^ and HCO_3_^−^ content, drives severe struvite-dominated scaling, progressively suppressing flux across cycles. Despite this severity, physical cleaning of the fouled membrane restored approximately 98–99% of the starting flux, confirming that the absence of compressive hydraulic pressure in FO preserves scaling reversibility, a practical advantage over pressure-driven processes. Visual MINTEQ correctly predicted the dominant mineral families, but the actual deposits were governed by kinetic effects. This reflects a limitation of equilibrium models, which capture thermodynamic supersaturation but not reaction rates or pathways. As a result, thermodynamically favoured minerals may be kinetically inhibited, allowing metastable phases to dominate. Thermodynamic modelling and experimental characterisation are therefore complementary: one defines the scaling driving force, while the other identifies the phases formed under FO interfacial conditions. High rejection of multivalent ions (Mg^2+^, Ca^2+^, PO_4_^3−^, and SO_4_^2−^) was maintained throughout, confirming that membrane integrity was preserved despite severe scaling. These findings indicate that feed hydrolysis state should guide FO operating conditions and cleaning strategies, and that simple hydraulic flushing is an effective, energy-efficient approach to controlling inorganic scaling. Future research should use real human urine and investigate the combined effects of inorganic and organic fouling. The performance of chemical cleaning under progressive multi-cycle scaling should also be evaluated. In addition, a more comprehensive assessment of ion rejection, including NH_4_^+^ and HCO_3_^−^, is required, together with BET surface area analysis to better describe deposit structure and porosity. The potential of pH adjustment of hydrolysed urine prior to FO treatment should also be explored as a scaling mitigation strategy, given the strong pH dependence of carbonate and struvite supersaturation in SHU. Finally, XRD and FTIR analysis are recommended to confirm the crystallographic identity of the observed mineral phases. Overall, this work provides a mechanistic basis for understanding and predicting inorganic scaling in FO-based urine treatment systems, offering guidance for operating conditions, cleaning strategies, and process scale-up toward sustainable nutrient and water recovery from source-separated urine.

## Figures and Tables

**Table 1:** Ionic composition and key physicochemical properties of SFU and SHU.

Properties	SFU	SHU
Na^+^ (mg/L)	2161	2110
K^+^ (mg/L)	1564	1560
Mg^2+^ (mg/L)	97	5
Ca^2+^ (mg/L)	160	15
NH_4_^+^ (mg/L)	0	4900
Cl^−^ (mg/L)	3546	3100
SO_4_^2−^ (mg/L)	1440	1440
PO_4_^3−^ (mg/L)	1940	1400
HCO_3_^2−^	0	17,400
pH	6.0	9.0
Ionic strength	≈0.17 M	≈0.4 M
Osmotic pressure (bar)	≈13	≈23

## Data Availability

The original contributions presented in this study are included in the article/Supplementary Materials. Further inquiries can be directed to the corresponding author.

## Supplementary Materials

The following supporting information can be downloaded at: https://www.mdpi.com/article/10.3390/membranes16060197/s1. Table S1: Composition of synthetic fresh urine (SFU) and synthetic hydrolysed urine (SHU) including chemical purity and suppliers. Table S2: Calculated saturation indices (SI) of mineral phases with positive saturation indices for SHU at increasing concentration factors, predicted using Visual MINTEQ (version 4.0). Table S3: Calculated saturation indices (SI) of mineral phases with positive saturation indices for SFU at increasing concentration factors, predicted using Visual MINTEQ (version 4.0). Refs. [1,2,3] are cited in the Supplementary Materials.
