# Supplementary material for: Inorganic Scaling Mechanisms During Forward Osmosis Concentration of Fresh vs. Hydrolysed Urine: Theoretical Modelling and Experimental Validation
Source: Membranes (Basel). 2026 Jun 5;16(6):197. doi: 10.3390/membranes16060197 (PMC13303410; doi:10.3390/membranes16060197)
Supplement: Supplementary file 1 [file membranes-16-00197-s001.zip › membranes-4237522-supplementary.pdf]

membranes

MDPI

Supplementary Information

Inorganic Scaling Mechanisms During Forward Osmosis Concentration of Fresh vs Hydrolysed Urine: Theoretical Modelling and Experimental Validation

Maano Tshimange [a], Sitabule Ṋamadzavho Enos [b], Judy Lee [a] and Siddharth Gadkari [a,*]

[a] School of Chemistry and Chemical Engineering, University of Surrey, Guildford, Surrey GU2 7XH, United Kingdom
[b] Sasol Research & Technology, Sasolburg 1947, South Africa
* Correspondence: author. Tel: +44 (0)1483 682676. E-mail: s.gadkari@surrey.ac.uk (Siddharth Gadkari)

Table S1: Composition of SFU and SHU [1-3].

	SFU	SHU	Purity (%)	Supplier
Parameters	Value			
pH	6	9		Sigma-Aldrich
Urea (g/L)	15	-	99.0–100.5	Sigma-Aldrich
Ammonium bicarbonate (g/L)	-	21.4	≥99.0	Sigma-Aldrich
Ammonium chloride (g/L)	1	1.74	≥99.0	Sigma-Aldrich
Citric acid (g/L)	0.92	0.92	≥99.5	Fisher Scientific
Creatinine (g/L)	2.67	2.67	98	Sigma-Aldrich
Glycine (g/L)	0.19	0.19	98.5	Sigma-Aldrich
Creatine (g/L)	0.16	0.16	≥98	Sigma-Aldrich
Hippuric acid (g/L)	0.97	0.97	98	Fisher Scientific
Uric acid (g/L)	0.61	0.61	≥99	Fisher Scientific
Sodium chloride (g/L)	2.57	2.57	99.5	Fisher Scientific
Sodium sulfate (g/L)	2.13	2.13	99	Fisher Scientific
Potassium chloride (g/L)	2.98	2.98	≥99.0	Fisher Scientific
Magnesium chloride hexahydrate (g/L)	0.81	0.04	99	Fisher Scientific
Sodium phosphate monobasic (g/L)	2.40	2.1	99	Fisher Scientific
Calcium chloride dihydrate (g/L)	0.59	0.04	99	Fisher Scientific

 https://doi.org/10.3390/membranes16060197

Table S2: Calculated saturation indices (SI) of mineral phases with positive saturation indices for SHU at increasing concentration factors, predicted using Visual MINTEQ.

	Concentration factor				
**Mineral precipitates**	**1**	**2**	**3**	**4**	**5**
Aragonite	1.322	1.664	1.855	1.989	2.093
$Ca_3(PO_4)_2$ (am1)	-1.367	-0.742	-0.413	-0.18	0
$Ca_3(PO_4)_2$ (am2)	1.404	2.029	2.358	2.591	2.771
$Ca_3(PO_4)_2$ (beta)	2.495	3.121	3.449	3.682	3.862
$Ca_4H(PO_4)_3{\cdot}3H_2O(s)$	1.037	1.922	2.375	2.693	2.937
$CaCO_3xH_2O(s)$	0.131	0.47	0.656	0.786	0.886
Calcite	1.468	1.81	2.001	2.135	2.239
Dolomite (disordered)	2.487	3.176	3.562	3.833	4.043
Dolomite (ordered)	3.598	4.287	4.672	4.944	5.153
Huntite	1.859	3.243	4.018	4.564	4.984
Hydroxyapatite	11.165	12.144	12.66	13.024	13.305
Magnesite	0.583	0.931	1.126	1.263	1.368
Struvite	1.585	-13.314	-13.038	-12.85	-12.709
Vaterite	0.896	1.237	1.428	1.563	1.666

Table S3: Calculated saturation indices (SI) of mineral phases with positive saturation indices for SFU at increasing concentration factors, predicted using Visual MINTEQ.

	Concentration factor				
**Mineral precipitates**	**1**	**2**	**3**	**4**	**5**
$Ca_3(PO_4)_2$ (am1)	-1.641	-1.062	-0.649	-0.326	0.705
$Ca_3(PO_4)_2$ (am2)	1.109	1.688	2.101	2.424	3.455
$Ca_3(PO_4)_2$ (beta)	1.779	2.358	2.771	3.094	4.125
$Ca_4H(PO_4)_3{\cdot}3H_2O(s)$	2.639	3.445	4.016	4.459	5.86
$CaHPO_4(s)$	1.119	1.351	1.515	1.642	2.039
$CaHPO_4{\cdot}2H_2O(s)$	0.83	1.058	1.218	1.341	1.72
Gypsum	-0.568	-0.382	-0.25	-0.148	0.175
Hydroxyapatite	8.202	9.127	9.787	10.303	11.958
$MgHPO_4{:}3H_2O(s)$	0.042	0.273	0.434	0.559	0.941

https://doi.org/10.3390/membranes16060197

References

1. Zhang, J., Q. She, V.W. Chang, C.Y. Tang, and R.D. Webster, *Mining nutrients (N, K, P) from urban source-separated urine by forward osmosis dewatering.* Environmental science & technology, 2014. **48**(6): p. 3386-3394.
2. Almuntashiri, A., A. Hosseinzadeh, U. Badeti, H. Shon, S. Freguia, U. Dorji, and S. Phuntsho, *Removal of pharmaceutical compounds from synthetic hydrolysed urine using granular activated carbon: column study and predictive modelling.* Journal of Water Process Engineering, 2022. **45**: p. 102480.
3. Ray, Hannah, F. Perreault, and T.H. Boyer, *Urea recovery from fresh human urine by forward osmosis and membrane distillation (FO–MD).* Environmental Science: Water Research & Technology, 2019. **5**(11): p. 1993-2003.

Disclaimer/Publisher's Note: The statements, opinions and data contained in all publications are solely those of the individual author(s) and contributor(s) and not of MDPI and/or the editor(s). MDPI and/or the editor(s) disclaim responsibility for any injury to people or property resulting from any ideas, methods, instructions or products referred to in the content.

https://doi.org/10.3390/membranes16060197
